# Foodways in transition: food plants, diet and local perceptions of change in a Costa Rican Ngäbe community

**DOI:** 10.1186/s13002-015-0071-x

**Published:** 2016-01-06

**Authors:** Ugo D’Ambrosio, Rajindra K. Puri

**Affiliations:** Laboratori de Botànica, Facultat de Farmàcia - Unitat Associada CSIC, Universitat de Barcelona, Av. Joan XXIII s.n., 08028 Barcelona, Catalonia Spain; Institut Botànic de Barcelona (IBB-CSIC-ICUB), Passeig del Migdia s.n., Parc de Montjuïc, 08038 Barcelona, Catalonia Spain; Centre for Biocultural Diversity, School of Anthropology and Conservation, University of Kent, Canterbury, UK

## Abstract

**Background:**

Indigenous populations are undergoing rapid ethnobiological, nutritional and socioeconomic transitions while being increasingly integrated into modernizing societies. To better understand the dynamics of these transitions, this article aims to characterize the cultural domain of food plants and analyze its relation with current day diets, and the local perceptions of changes given amongst the Ngäbe people of Southern Conte-Burica, Costa Rica, as production of food plants by its residents is hypothesized to be drastically in recession with an decreased local production in the area and new conservation and development paradigms being implemented.

**Methods:**

Extensive freelisting, interviews and workshops were used to collect the data from 72 participants on their knowledge of food plants, their current dietary practices and their perceptions of change in local foodways, while cultural domain analysis, descriptive statistical analyses and development of fundamental explanatory themes were employed to analyze the data.

**Results:**

Results show a food plants domain composed of 140 species, of which 85 % grow in the area, with a medium level of cultural consensus, and some age-based variation. Although many plants still grow in the area, in many key species a decrease on local production–even abandonment–was found, with much reduced cultivation areas. Yet, the domain appears to be largely theoretical, with little evidence of use; and the diet today is predominantly dependent on foods bought from the store (more than 50 % of basic ingredients), many of which were not salient or not even recognized as ‘food plants’ in freelists exercises. While changes in the importance of food plants were largely deemed a result of changes in cultural preferences for store bought processed food stuffs and changing values associated with farming and being food self-sufficient, Ngäbe were also aware of how changing household livelihood activities, and the subsequent loss of knowledge and use of food plants, were in fact being driven by changes in social and political policies, despite increases in forest cover and biodiversity.

**Conclusions:**

Ngäbe foodways are changing in different and somewhat disconnected ways: knowledge of food plants is varied, reflecting most relevant changes in dietary practices such as lower cultivation areas and greater dependence on food from stores by all families. We attribute dietary shifts to socioeconomic and political changes in recent decades, in particular to a reduction of local production of food, new economic structures and agents related to the State and globalization.

## Resumen

**Antecedentes:**

Las poblaciones indígenas están viviendo rápidas transiciones etnobiológicas, alimentarias y socioeconómicas mientras se integran en sociedades modernizantes. Para entender mejor la dinámica de dichas transiciones, este artículo caracteriza el dominio cultural de las plantas comestibles y analiza sus relaciones con la dieta actual, y las percepciones para los cambios señalados por parte de los Ngäbe del sur de Conte-Burica, Costa Rica, mientras la producción de plantas alimentarias por parte de sus residentes se hipotetiza estar en drástica recesión con un descenso del área productiva y la implementación de nuevos paradigmas de conservación y desarrollo.

**Métodos:**

Se realizaron listas libres extensas, entrevistas y talleres grupales para recolectar los datos entre 72 participantes sobre conocimiento de plantas comestibles, prácticas actuales alimentarias y percepciones de los cambios, mientras que se llevó a cabo análisis de dominios culturales, análisis estadísticos descriptivos y desarrollo de temas fundamentales para la interpretación de la información.

**Resultados:**

Los resultados muestran un dominio de plantas alimentarias compuesto de 140 especies, de las cuales 85 por ciento crecen localmente, con un nivel de consenso cultural medio, y cierta variación asociada a la edad. A pesar de que muchas plantas aún crecen en el lugar, en muchas de las especies clave se observó un retroceso en el abastecimiento local y hasta el abandono total. Es por ello que el dominio parece ser en gran medida teórico, con poca evidencia de su uso; y la dieta hoy en día es predominantemente dependiente de comidas compradas en la tienda (más del 50 % de ingredientes básicos), muchas de las cuales no fueron sobresalientes o hasta reconocidas como plantas comestibles. Mientras que la mayoría de los cambios en la importancia de las plantas comestibles fueron atribuidos a cambios en preferencias culturales hacia productos procesados comprados en la tienda así como cambios en valores asociados con la agricultura y la auto-suficiencia alimenticia; los Ngäbe son mayoritariamente conscientes de los cambios en las actividades de subsistencia familiares, y la subsiguiente pérdida de conocimiento y uso de las plantas comestibles, siendo promovidos por cambios políticos y sociales, a pesar del incremento en cobertura boscosa y biodiversidad.

**Conclusiones:**

Los hábitos agroalimentarios entre los Ngäbe están cambiando de formas diversas y de algún modo desconectadas: el conocimiento de plantas comestibles es variado, reflejando algunos de los cambios en las prácticas alimentarias, tales como menores áreas productivas y mayor dependencia en productos comprados en el comercio por parte de todas las familias. Atribuimos las transformaciones alimentarias a cambios socioeconómicos y políticos en décadas recientes, en particular a una reducción de la producción local de comida, nuevas estructuras y agencias económicas relacionadas con el Estado y la globalización.

## Background

Indigenous populations, such as the Ngäbe in Costa Rica, are undergoing rapid ethnobiological, nutritional and socioeconomic transitions while being increasingly integrated into globalizing and modernizing societies [1–3]. To better understand the dynamics of these transitions, especially how they interact with one another, this article analyses change in the cultural domain of food plants (*krigä kwedäga*), changes in culinary practices, and local perceptions of these changes among the Ngäbe people of Southern Conte-Burica. Our findings suggest that knowledge and practice in Ngäbe foodways are more varied than in the past, while explanations of change offered suggest a complex set of intersecting drivers of change. The study also contributes detailed ethnographic and ethnobotanical data to a greatly understudied area of Central America [2].

As of 2011, at least nine different pre-Columbian ethnic groups and more than 100,000 people self-identified as indigenous live in Costa Rica, representing 2.4 % of the total population. More than half live in the 24 distinct indigenous territories which cover 8 % of the country, mostly in its southern part, while the other half lives outside of them. The Bribri and Cabécar are the most numerous (with about 20 % of the total indigenous population each), followed by the Chorotega in the Northwest and the Ngäbe (including the distinctive Buklé) in the South, (about 10 % of the total indigenous population each). The least numerous include the Brunka, the Huetar, the Teribe and the Maleku, each with 5000 members or less [4]. Except for the Chorotega, which have Oto-Mangue ancestry, all are of Chibchan descent [5]. The degree of acculturation is prominent in most cases, being higher closer to urban areas or in the lowlands, and predominantly amongst the youth. Only five out of nine indigenous languages are spoken today, but even these are vulnerable with respect to Spanish, especially for two of them, Maleku and Brunka, which are highly threatened as only a few fluent speakers remain [5]. It is worth mentioning that in the last census in 2011, the Buklé were newly lumped together with the Ngäbe into the Guaymí ethnicity [6], but this classification is disliked by both groups, and in fact reduces the usefulness of the census, in terms of its ability to monitor vulnerable groups.

Ngäbe populations, mostly dwelling in four forested and semi-forested territories of southern Costa Rica, are descendants from Panamanian migrant populations arriving since the early 20^th^ century [3]. A process of economic and livelihood diversification amongst families has taken place over the last two to three decades, whereby food plants originally obtained from local ecosystems and cultivated fields—especially rice (*Oryza sativa*), beans of several genera (*Phaseolus*, *Cajanus*, *Vigna*), maize (*Zea mays*), and various underground storage organs (*Dioscorea* spp., *Manihot esculenta*, *Xanthosoma* spp.) —are now produced and consumed in lesser quantities, or are being substituted by commercial versions bought from outside, implying significant transformations and new scenarios in Southern Conteburican ethnobiological affairs, from crop production and consumption to food preparation and exchange.

Regrettably, thorough ethnobiological, agroculinary or ethnographic studies of Chibchan Amerindian groups such as the Ngäbe are very limited, both in general terms and specifically dealing with local production of food plants, foodways, and their drivers of change. This is especially apparent when compared to other Central American macrolinguistic families, such as the Mayas [7], or from other parts of the world (e.g., North America or Brazil). Besides some governmental and non-governmental technical reports and educational materials, academic literature on Costa Rican indigenous territories is limited (see below). Nonetheless, the Ngäbe are the most numerous indigenous group of Lower Central America [8], as well as the largest among the dozen other extant languages within the Chibchan macrolinguistic family [9].

To date, the most detailed ethnography on the Ngäbe people is the late Philip D. Young’s classic work *Ngawbe: Tradition and Change among the Western Guaymí of Panamá* [10], though a more recent reflexive ethnography undertaken also in Panamá by Alexis Karkotis [11], *Now we live together,* does present a more up-to-date analysis of change in the area. In relation to more ethnobiologically-oriented works in Chibchan populations, research is very limited and shallow; no in-depth systematic studies have been made. Academic journal articles include the work of Camacho-Zamora where a description of uses of plants by Cabécar communities is given [12], and Hazlett’s study on the ethnobotany of Ngäbe and Cabécar settlements in Costa Rica, which describes knowledge of the uses of plants collected, and hypothetical reasons for differences observed amongst communities [13].

In addition to these descriptive works, Madriz published on the uses of wild food plants in tropical forests in the Tayní indigenous territory in Costa Rica, inhabited mostly by Cabécar [14]. After a listing of plants and uses, Madriz proposes further research, most of which relates to agroforestry and food security; no mention is made about other factors (economic, social, political) that might be driving change, nor the opinions, aspirations or beliefs of local people. Along these lines, Zaldivar et al. present a more comprehensive analysis of the species diversity of homegarden edible plants in two Costa Rican indigenous communities (the Bri-Bri in Talamanca and the Ngäbe in Coto Brus) [15]. They examine contemporary use of food plants in 138 homegardens from an ecological point of view, calculating diversity indices, community similarities and differences in the presence of food plants, as well as the potential for in situ conservation of genetic diversity. In 2007, Castañeda and Stepp published an article looking at the ethnoecological importance value (EIV) methodology to assess the cultural importance of ecosystems as sources of useful plants for the Ngäbe of Costa Rica [16]. They found that to the Ngäbe of Coto Brus, mature forests and their edges were the most culturally valuable successional stages, in terms of sources of wild edible plants. Early secondary growth and older secondary forests and their edges were of minor importance. Most of the information in Castañeda’s and Stepp’s article comes from Castañeda’s master’s thesis [17], where cultural domain analysis (CDA) is carried out in order to study elicited wild food plants in Coto Brus. Moreover, in Camacho’s publication “20^th^ century’s frontiers: the exclusion of the Guaymí in Costa Rica” [3], an entire section is devoted to Conte Burica’s staple production in the year 1993, contrasting 20 different households, half in the southern section of the territory and half in the north. Camacho’s work constitutes the most recent estimate of Conte Burica’s staples production in the literature, while also considering the significance of historical aspects and concerns for the future.

Koshear, in her PhD thesis entitled “Guaymí agriculture, forest utilization and ethnobotany in Coto Brus (Costa Rica): an analysis of sustainability”, collected ethnobotanical, agricultural, and NTFPs information with the Ngäbe in the Coto Brus Indigenous Reserve [18]. Koshear gives useful historical and ethnographic information, basic crop production estimates, detailed plant and animal lists, land tenure aspects and issues, as well as an analysis of sustainability and attitudes towards the environment, especially relevant to the current research reported here. Mostly descriptive in character, this work is relevant for the understanding and analysis of natural resource sustainability, although at that time development and conservation programs had just recently started. Other researchers including Gordon [19], Joly et al. [20, 21], Bletzer [22] and Border [23] have conducted studies which include ethnobotanical information relating to medicinal plant use by the Ngäbe of Panamá, mostly from Bocas del Toro province (known as *Nö kribo* in Ngäbere). Bletzer includes ethnographic information on local healers and illnesses they treat [22]. Border's Master's thesis entitled "Ethnobotany of the Ngäbe People of Panama" from 2011, is a description of 160 medicinal plants elicited by local healers (*sukias*) [23]. Three other works involving the Ngäbe from Panama are worth mentioning here: Pastrana [24], Pastrana et al. [25], and Méndez et al. [26], each with a preponderant agroforestry focus, where the arboreal component of Ngäbe agroforestry systems is analyzed, showing the relevance of these systems from multiple ecological and productive perspectives. Another pertinent work is Montero and Corrales' study of non-traditional edible leaves, flowers and stems in Costa Rica [27]. Other various ethnobiological works in the area include a related master’s thesis looking at wild fauna use and abundance [28], a few technical reports [29], unpublished ethnobotanical fieldwork carried out by various undergraduate students [30] and a book on Ngäbe ethnobotanical knowledge in Costa Rica [31].

Despite this rich and wide-ranging research about indigenous communities in Costa Rica, the dynamics of food plants, foodways and their transformations has been neglected. Therefore, this article seeks to examine such dynamics through an analysis of food plants, their changing role in diets and local explanations for recent changes in their importance. We have set out to answer three main research questions: What is the composition and structure of the cultural domain of food plants amongst the Ngäbe in Conte-Burica? For the most salient of food plants, what are their ethnobotanical characteristics? Finally, what changes have happened to the local production of these most salient edible plants over the last two to three generations, as recalled by the Ngäbe? Much of the research was descriptive in nature, but to the extent that we attempted to uncover and develop an emic model for change in foodways, our guiding hypothesis derives from the ethnobotanical implications of the *nutritional transition* concept [32], a process of dietary change from traditional foods (low fat, low sugar, high fiber and highly diverse) to a reliance on a ‘western diet’ (high calories and fat, low diversity), due to demographic shifts (mainly population growth and urbanization), introduction of a market economy, and cultural changes associated with the spread of Western values (globalization) [33, 34].

Following a description of the methods used, we first describe findings from freelist elicitations supplemented by data from structured and group interviews and inventories concerning the cultural domain of food plants in Conte-Burica as a whole, as well as detailing the ethnobotanical characteristics of those folk species considered most salient. We then analyze perceived changes in local production and dietary provisioning of these most salient food plants. We conclude with a discussion of the interrelations of various transitions and transformations taking place in Ngäbe foodways.

## Methods

### Southern Conte-Burica

**Fig. 1:**
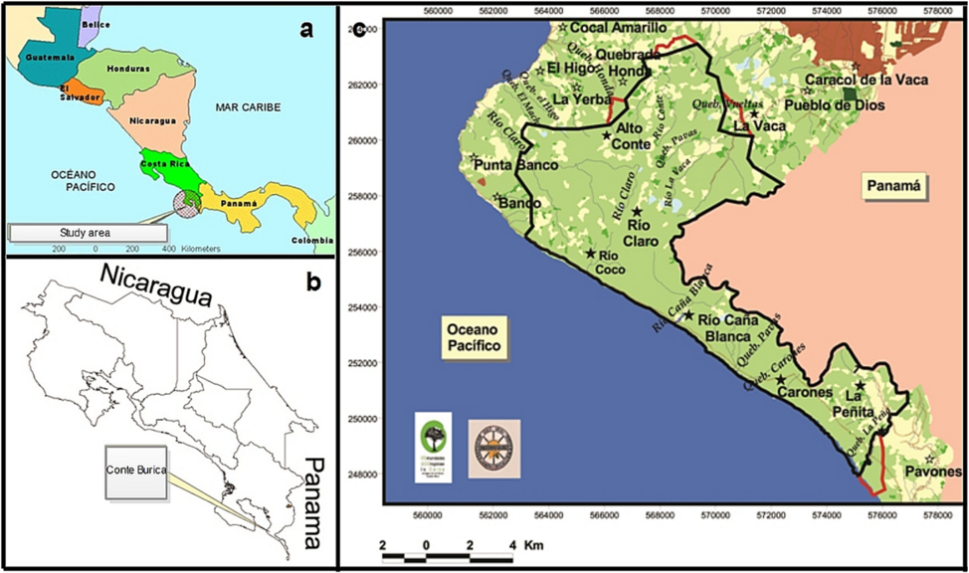
Study area. **a** Central America south of the Yucatan Peninsula. Source: [66]; **b** location of Conte-Burica in Southern Costa Rica. Source: [63]; **c** Conte-Burica indigenous territory map. The black line corresponds to current limits, and the red line to suggested extensions. Southern Conte-Burica is separated from its Northern part by the Caña Blanca River. Source: [67]

The Burica Peninsula, on the Pacific side of the Central American Isthmus (Fig. 1a and b), is an area of land stretching between the Dulce Gulf to the west, and the Charco Azul Bay (or Chiriqui Gulf) to the east, and at present corresponds to a territory shared between Panama to the east and Costa Rica to the west.

Within the Costa Rican western side of the Peninsula (Fig. 1c), the indigenous territory of Conte-Burica has a surface area of about 12,000 ha (120 km^2^), although, according to different sources 40–60 % of their land is still in hands of non-indigenous owners [35, 36]. Conte-Burica is a transborder territory with two distinct areas clearly established by the Caña Blanca River. The Northern section of the territory is larger in area, easily accessible from Costa Rica (via Laurel and Conte-Abajo), more densely populated, less forested, closer to towns and governmental institutions, and also maintains better infrastructural conditions. The Southern region (south of Caña Blanca), considered here, is less populated, has suffered less deforestation and is more easily accessible from Panamá (via Puerto Armuelles or Limones), but difficult to access in the rainy season. No settlement in all of Conte-Burica contains more than 350 inhabitants.

**Fig. 2:**
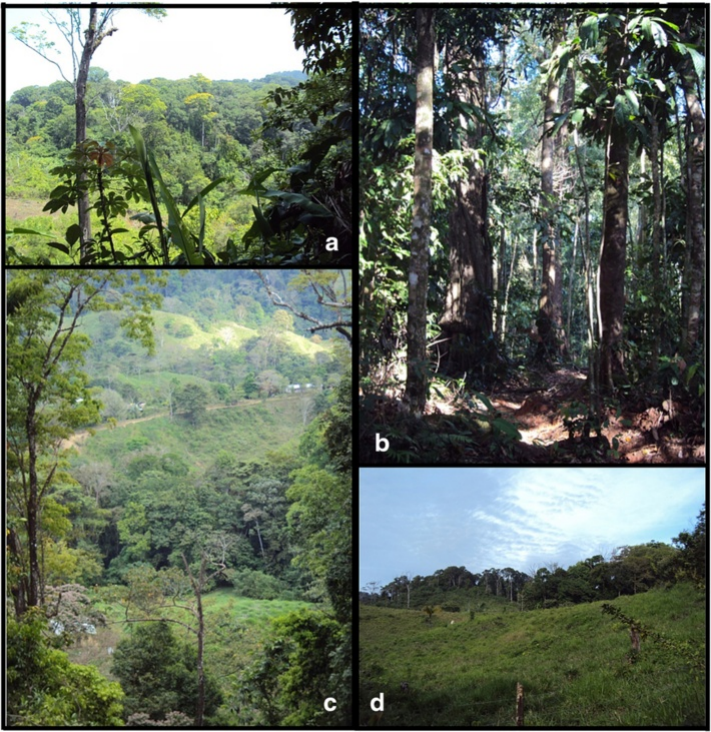
Local agroecosystems. **a** & **b** Lowland evergreen tropical forests are common in Southern Conte-Burica; **c** & **d** Amid forested areas, patches of grassland of different sizes can be found

Highly biodiverse tropical forests dominate while other vegetation formations thrive in this area; outside the territory, significantly deforested agricultural areas are found. The forests are classified as dense tropical broad-leaved evergreen well-drained lowland forest, while small areas of cloud forest exist in the more highly elevated premontane areas [37] (Fig. 2a and b). In addition, smaller patches of tropical evergreen lowland shrub-land, and tropical lowland well-drained grasslands with some trees can be found (Fig. 2c and d). One report of 2000 claims that 69 % of the territory was covered by forests [38], a second report cites 64 % as forested area [37], while a third 52 % [39]. These discrepancies are probably linked to the inclusion or exclusion of patches of old African oil palm plantations in calculations of total forested area, in addition to lack of field data.

According to estimations from the latest census from 2011, Conte-Burica has a population of about 2000 inhabitants [4], while in the year 2000 there were roughly 1000 residents [6]; thus the population has virtually doubled in the last decade. Almost 40 % of inhabitants in the territory are non-indigenous [4], coming from various parts of Costa Rica and settling mostly in the northern section of the territory. Prior to two to three decades ago, Conteburicans based their economy and diet on subsistence agriculture of basic grains (*Oryza sativa, Phaseolus vulgaris, Zea mays*), along with the sale of surplus produce, complemented by hunting, fishing and gathering of forest products [3, 28]. The region transformed socioeconomically with the proliferation of banana plantations in the 1960’s, leading to wage labor of young and adult Ngäbe men in neighboring banana farms and later on, palm oil plantations [3, 40]. Currently, most of the region has been socioeconomically weakened due to unemployment, especially since farms and plantations started to close in the 1980's [41]. The character of Conteburican economy today (as well as other indigenous territories in the country) can be seen as a combination of subsistence and monetary contributions, in what is known as a mixed-economy. A part of this cash (from wage labor, government jobs, or welfare income), is subsequently re-invested in subsistence production, with a quantity of it being used for store-bought provisions and supplementary commodities not directly associated with subsistence, such as mobile phones or televisions. Only a much reduced number of families (those managing communal funds of great quantities or a very few individuals involved in sporadic drug smuggling through the Pacific shore within the territory) have the possibility to accumulate high amounts of money. In many cases, money is spent as it comes and when it is totally spent, families have to resell locally those assets at much lower prices.

### Data collection and analysis

**Fig. 3:**
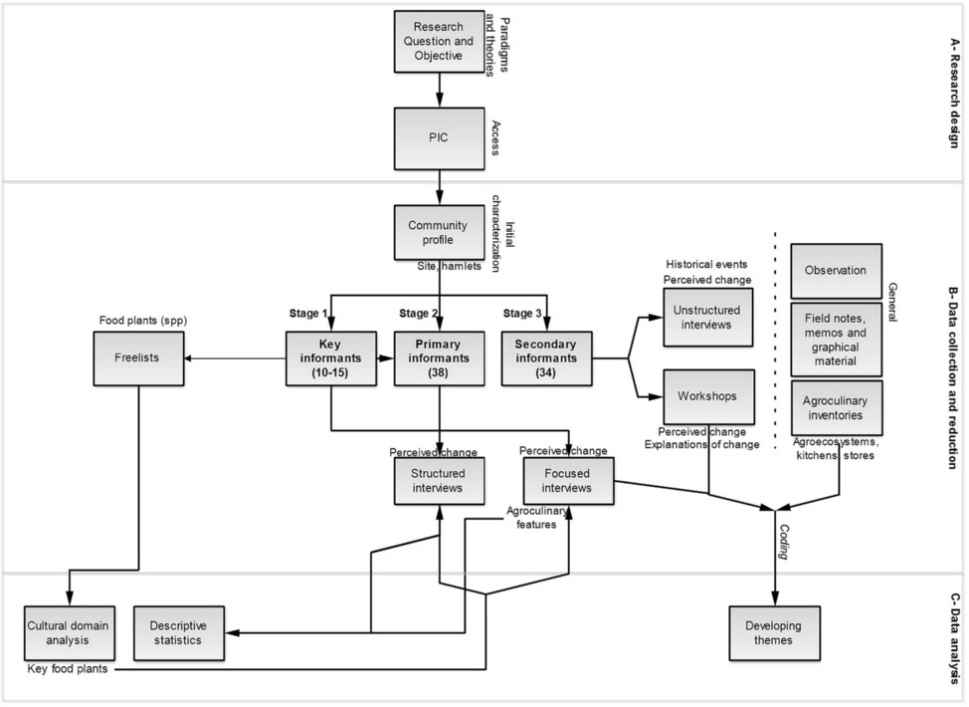
Overall ethnographic methods employed. Methods employed for research design, data collection and analysis of food plants and perceived production changes amongst most salient plants in Conte Burica, Costa Rica

Results presented in this article are drawn from a larger research project undertaken at the University of Kent (UK) and Costa Rica between the years 2009 and 2013, on change in agricultural and culinary systems of the Ngäbe-Buglé indigenous territory of Conte-Burica, Costa Rica [2]. Fieldwork in Southern Conte Burica took place between 2010 and 2012, totaling twelve full months of data collection. Data collection methods were employed with 72 participants (28.8 % of total local population) from 32 distinct households (41.5 % of total households in the area of study), including: successive freelisting of edible plants; unstructured, focused and structured interviews about food plants and perceived changes in local production; workshops and group activities; food inventories (in agroecosystems, kitchens and local stores); and, prolonged observation (Table 1, Fig. 3). According to methods used and time spent, participants were subdivided into primary (*n* = 38) and secondary (*n* = 34). Primary informants included 10 key informants, with whom longer times were spent.

**Table 1:** Sampling and informant typology (older than 10 years old), according to number of participating individuals and households

	Individuals	Households	Techniques used
	(% of total)	(% of total)	(topics covered)
Key informants	15	13	Extensive freelists
(included within primary informants)	(7 %)	(*15* %)	(food plants)
			Focused interviews
			(qualitative perceived changes in local production of key food plants)
Primary informants	38	13	Structured interviews
(incl. key informants)	(15.2 %)	(*15* %)	(quantitative perceived changes in local production of key food plants)
Secondary informants	34	22	Group interviews
	(13.6 %)	(26.5 %)	Unstructured interviews
			(key food plants & qualitative perceived changes for both techniques)
Current sample	72	35	
	(28.8 %)	(41.5 %)	
Total population in the study area	250	85	
	(100 %)	(100 %)

Prior informed consent was obtained verbally before interviews and other activities were conducted, with the aims, methodology, and outcomes of the study being explained to all participants. We followed ethical guidelines adopted by the International Society of Ethnobiology (2006) and research was evaluated and approved by the Ethics Committee of the School of Anthropology and Conservation at the University of Kent at Canterbury, UK. Due to previous experiences with various national and local institutions, who express fears of extraction of local natural resources by outsiders, during initial phases of PIC it was agreed not to collect all plant specimens but only those problematic to identify. Many species were cultivated and easily recognized in the ground. When specialists’ identifications were deemed necessary, and sufficient vegetative plant material was available, botanical samples were collected, pressed and dried in the field (following local, national and international guidelines and regulations), and identified at the Juvenal Valerio Rodríguez Herbarium (for vegetative material) or at the Agronomy Seed Bank (for the case of seeds), both at the National University of Costa Rica (UNA) in Heredia. Due to this limitation, five edible plants were not identified, representing less than 4 % of the total of food plants elicited.

*Successive freelists* [42] were employed with key informants (*n* = 10) during the initial stages of research, to determine the potential pool of edible plants known by people [43, 44]. The first 10 participants (over 35 years old) were selected for their willingness to participate, in order to garner an initial yet thorough list. Participants included Ngäbere speakers and non-speakers, women and men, with different socio-economic and cultural characteristics. They were asked to list all the plants they could think of, that could be eaten and/or drunk, including roots, leaves, fruits, seeds, condiments, or any other plant-derived food, both wild and cultivated. The question was refined following initial elicitation from participants in order to generate a comprehensive list of items [44]. We used cultural domain analysis [43, 44], to analyze data from freelists, particularly to investigate consistency of the domain, frequency of items listed, rank order as well as saliency, key items derived from the analysis, and a preliminary description of intracultural variation. ANTHROPAC software [45] was used to analyze the freelist items. Smith’s S, a measure of cultural salience, was calculated for all items listed [43, 46]. This is a weighted average of the (inverse) rank of an item across multiple free-lists, where each list is weighted by the number of items in the list. The freelists provided the baseline data for designing further focused, structured and group interviews by establishing key species to emphasize in further stages of the research as well as the preliminary characteristics of their production and consumption [44].

**Fig. 4:**
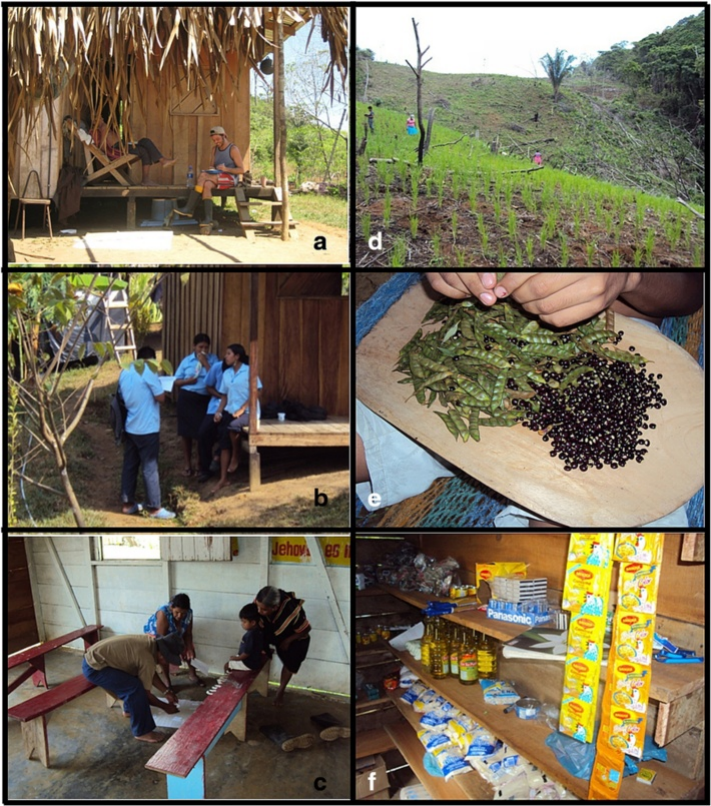
Research methods for data collection. **a** Focused interview; **b** Autoethnography; **c** Workshop; **d** Agricultural inventory; **e** Culinary inventory; **f** Store inventory

To gain additional descriptions of past and current foodways, two different interviews were conducted during initial stages of research to complement the analysis of present culinary repertoires and perceived change in local production of key food plants [47, 48]. These interviews were conducted in a broad range of natural settings—in people’s gardens, cultivated fields, kitchens, at dining tables, in local schools, during celebrations, walking through forests and trails, or travelling in and out of the territory. Open-ended questions were generally asked in the same order, but interviewees were given freedom to speak freely on tangential subjects should they wish, a common occurrence with any interview-based data collection method. *Unstructured interviews* with 10 thematic open-ended questions were undertaken with eight secondary informants. Lasting approximately an hour each, these involved gathering general information on current agroculinary systems and their perceived transitions. *Focused interviews* were conducted with 10 key older informants (those participating in initial freelists) to collect data regarding key folk species, their agroculinary characteristics within local food systems, and additional detailed information and clarification of the explanations of agroculinary change stipulated in freelists (Fig. 4a). Agroculinary information derived from these interviews was transcribed, manually coded and analyzed by seeking major themes to describe foodways transitions.

*Structured interviews* on perceived change in local production were carried out with all 38 primary informants in a later stage of research. Being based on rating scales commonly used in social sciences’ questionnaires and adapted to the aims of the intended research these interviews explored perceived change in local provisioning of food plants amongst different households and individuals [49, 50]. These included the 25 key folk species derived from the freelist data. Lasting less than an hour each, structured interviews focused on edible plants locally produced or provisioned by the household; it explicitly excluded those brought from outside Southern Conte Burica. Past household production was established from approximately 30 years ago (i.e., mid 1980's), or as better understood by informants one to two generations ago. First, the person was asked if he/she knew or did not know the food plant in question (both giving the name in Ngäbere and Spanish). If not known, the item was considered in the unknown category (U = 0) and we moved on to the following plant in the list. If the plant in question was known, five possibilities were recorded:

- Old produce, abandoned, disappearing (O = 1): Not produced/provisioned anymore (perceived as old plant, locally lost);
- Less, decreased (L = 2): Locally produced/provisioned more in the past than in the present (perceived as decreasing);
- Equal, no change (E = 3): Locally produces/provisions more or less equal now than in the past (perceived to remain equal);
- More, increased (M = 4): Locally produces/provisions more in the present than one-two generations ago (perceived as increasing);
- New (N = 5): Only of recent local production/provisioning (perceived as a new plant, locally new).

Structured interviews were analyzed using EXCEL (Microsoft 2007) and SPSS (IBM 2010) software to obtain basic descriptive statistics on perceived changes in key food plants such as their proportions, variation and relationships within food plants, as well as to generate graphical representations of the data.

To augment data collected with individual primary informants, three group interviews with 26 additional participants from 16 households were organized (considered as secondary informants), where socioeconomic and agroculinary issues were discussed. Two of these group activities were carried out with high school students (Fig. 4b); the remaining one was carried out with adults over two different mornings (Fig. 4c). Detailed notes were kept on both content and context of meetings (e.g. group dynamics, non-verbal responses), as well as graphic materials of several activities. The first workshop with high school students was devoted to exploration of declarative knowledge on food plants, discussion of current foodways, and their perception of changes within these systems. In a second group activity at the school, 12 students were initially trained to conduct semi-structured interviews and basic field inventories. They were asked to perform several interviews and inventories on food systems, as a way of promoting autoethnographic initiatives. The group interviews with adults served for discussion of present and past agricultural systems including food plants, varieties, and agroecosystems. Socioeconomic, historical and agroculinary information derived from workshops and autoethnography was coded and analyzed by identifying major themes used to describe foodways transitions in production stages.

Qualitative inventories partaken by the researchers supplemented the data on agroecosystem composition and structure (Fig. 4d), culinary elements (Fig. 4e), and store-bought foods (Fig. 4f). These agroculinary inventories (primarily listings of things) were carried out in several agroecosystems, kitchens, as well as in stores within the territory or the closest town of Puerto Armuelles throughout the whole research while being in the area by taking note of any additional plant-based food consumed and not reported by freelists participants and interviewees (e.g., various products containing *Triticum* flour or *Elaeis guineensis* oil). Qualitative inventories allowed supplementing the record of food elements given by informants, until exhausting the total set of food plants being consumed. Last but not least, prolonged observation and participant observation complemented other data collection procedures.

For consistency, simplicity and uniformity, ethnobotanical units (food plants) are named in their first appearance in English and Ngäbere, including scientific and for most cases Spanish names. For most common and recurrent species such as rice (*aro*, *Oryza sativa, arroz*) or coffee (*kabe*, *Coffea arabica, café*), names may be presented only in scientific form.

## Results

The domain investigated, *krigä, kriblü, krigwä btä krigwädri kwedäga* (and variations) embraces the Ngäbe ensemble of food plants; it is translated as the different plant parts (*krigä* for leaves, *kriblü* for flowers, *krigwä* for fruits and *krigwädri* for subterraneous organs) that can be eaten (*kwedäga*). To shorten we use here the term ‘*krigä kwedäga’* which corresponds to what we identify as edible/food plant. Botanical elements were recorded in Spanish and/or Ngäbere, according to the informant's choice. Language correspondence of unknown items was pursued with the most knowledgeable participants (elder Ngäbere-Spanish speakers) or the help of a local translator. Slight differences between botanical and local systems of classification are the rule. Thus, a distinction needs to be recognized between botanical taxa (species set by the International Association for Plant Taxonomy and agreed upon within the international scientific community) and folk taxa (categories at the species level as understood by local inhabitants of an area, usually agreed upon at a local or regional scale). In addition, as a few plants have disappeared in the area or are very hard to find, their unequivocal identification was impossible. How does one study something that has been already lost or that very few people know about? Although it may be impracticable to study loss directly, we can learn a great deal indirectly from synchronic variation, as shown later.

### Composition and structure of the edible plants’ domain (*krigä kwedäga*)

**Fig. 5:**
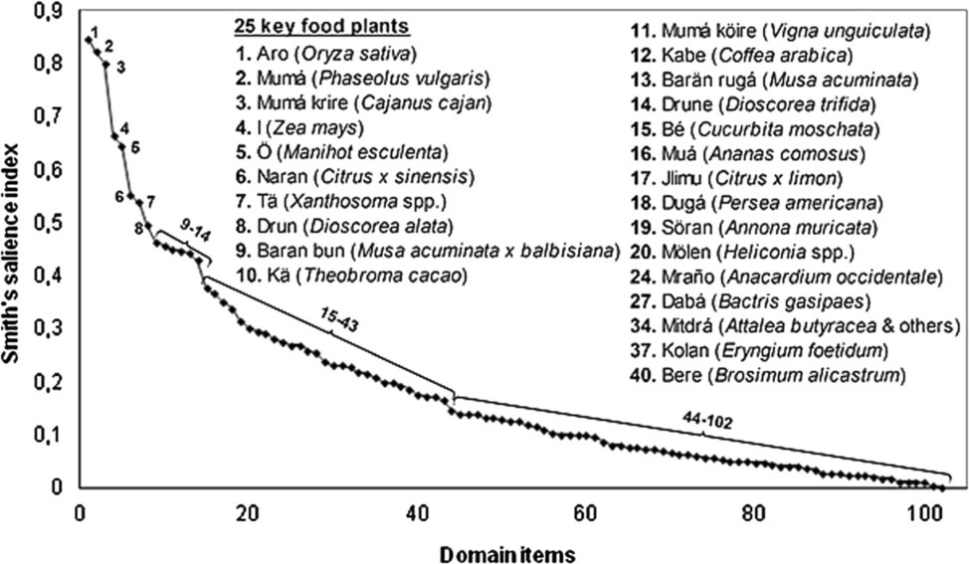
Smith's salience index curve with 102 items elicited in freelistings. Included are 25 key food plants and their correspondent ranking (In the list, notice that after item 20, ranking of key food plants does not match with Smith’s salience index, as species with lower S may be key, while others with higher S may not). Unclear 'elbow' between items 9 and 14

Following are the findings concerning the botanical elements constituting the cultural domain of food plants in the study area. Through extensive freelists with key informants, a total of 102 items were elicited (Appendix 1). Based on the cultural consensus analysis in ANTHROPAC, 25 items (24.5 %) are considered as fitting the consensus key (Fig. 5) [51], that is they are the ones that are most salient of the domain, and thus most likely to be listed by a typical member of this cultural community.

When plotting Smith’s saliency values (S) in a decreasing order (Fig. 5), a clear 'elbow' (indicating a distinction between the core most salient items in the domain and the rest) is not observed. Nevertheless, an apparent change in slope occurs after the eighth most salient item (*Dioscorea alata*), and after items 9 to 14 (*Musa A × B* to *Dioscorea trifida*), when the graph keeps decreasing its slope gradually, without significant changes. With a medium pseudo-reliability value of 0.793, and eigenvalue ratio, 1^st^ to 2^nd^ Factor, above 3 (3.512), the analysis indicates that amongst key informants the cultural domain of *‘krigä kwedäga’* is quite robust (medium consensus and single culture origin of informants). Freelist data is summarized in Table 2, showing total species elicited, freelist length range, mean and standard deviation per individual, pseudo-reliability, eigenvalue ratio, as well as folk species fitting the consensus key in the decreasing Smith’s salience index [52]. ANTHROPAC [45] was used to generate these results.

**Table 2:** CDA results applied to food plants' freelists (folk taxa). (*n* = 10 key informants)

Parameter	Value
Initial number of folk taxa elicited	102
Range of items elicited per informant	25–51
Mean freelist length per informant (±SD)	37.6 ± 10.7
Pseudo-reliability	0.793^a^
Eigenvalue ratio between first and second factor	3.512^b^
Number of folk taxa fitting the consensus key	25
(Percentage of total)	(24.5 %)
Folk taxa fitting the consensus key (=“key food plants”)	Aro, Mumá, Mumá krire, I, Ö, Naran, Tä, Drun, Baran bun, Kä, Mumá köire, Kabe, Barän rugá, Drune, Bé, Muá, Jlimu, Dugá, Söran, Mölen, Mraño, Dabá, Mitdrá, Kolan, Bere
(In decreasing saliency)	

^a^: Indicates medium consensus amongst informants. ^b^: Indicates single culture origin of informants

*Aro* (rice, *Oryza sativa*), *mumá* (common beans, *Phaseolus vulgaris*) and *mumá krire* (pigeon beans, *Cajanus cajan*) correspond to the three most salient food plants, with S indices higher than 0.8. *Ï* (maize, *Zea mays*) and *ö* (cassava, *Manihot esculenta*) follow, with values between 0.6 and 0.7. *Naran* (orange, *Citrus × sinensis*), *tä* (arrowleaf, *Xanthosoma* spp.) and *drun* (yams, *Dioscorea alata*) come after, with values between 0.5 and 0.6. A significant decrease in S value is observed in the following items: from *baran bun* (plantains, *Musa A × B*) to *drune* (yampee, *Dioscorea trifida*), with S values between 0.4 and 0.5. The remaining 88 items have S indices below 0.4.

In combination with, and complementing freelist data, another 38 folk taxa were added to the list of food plants collected through other means in later stages of the project. (A comprehensive list of food plants including all recorded folk species can be found in Appendix 2). Of the 140 botanical elements composing the current culinary repertoire in Southern Conte Burica, 99.3 % were plants (98.6 % angiosperms and 0.7 % pteridophytes), and another 0.7 % were fungi, indicating most knowledge about edible angiosperms and minor knowledge about fungi and other groups. Eudicots (72.2 %) dominated over monocots (27.8 %), while no gymnosperms, algae, or other *Plantae* groups were reported. Many of these plants are polyvalent, both within the domain of food plants, as well as between different domains. Hence, the same plant can have completely different preparations and uses when consumed by humans (e.g., *Zea mays* for making *tamale* or *chicha,* and *Manihot esculenta* leaf or root) and can also be utilized as animal food (e.g., *Zea mays*, *Manihot esculenta*, *Xanthosoma* spp., and *Musa* spp.), as both construction and utensil material (mostly trees and palms), for medicinal purposes (e.g., *Zea mays*’ silk, *Anacardium occidentale*’s bark), as firewood (*Persea americana*, *Inga* spp.) and/or used symbolically in social and cultural life (e.g., *Theobroma cacao* and *Zea mays*’ *chicha*).

**Fig. 6:**
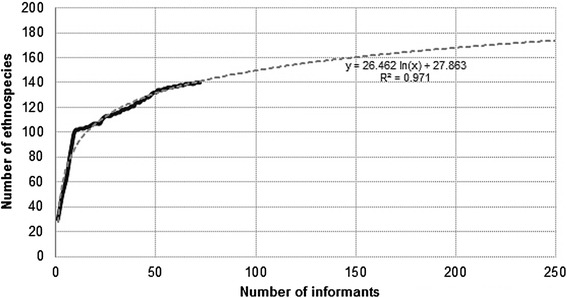
Informant-food plant curve (*n* = 72 informants). The relative change in slope observed in the figure at about 100 ethnospecies mentioned, corresponds to the last adult informant participating in extensive freelisting, while additional species were recorded by other means. In black: Observed values. In gray, dashed: Best-fitting theoretical curve (y = 26.462 ln x + 27.863; R^2^ = 0.971)

The informant-species curve shown in Fig. 6 was constructed from species lists obtained from all 72 informants using the methods previously described.

The logarithmic function estimates that 80.5 % of a potential maximum of 174 species composing the domain of edible plants (*‘krigä kwedäga’*) have been recorded during this study. This indicates that sampling method and size were adequate, and also demonstrates that a mixed methods approach is particularly useful in contexts of this sort. According to these estimates, by doubling the number of informants, only twenty additional plants would have been recorded. Moreover, the number of taxa and categories provides a solid representation of the cultural domain containing the culinary repertoire in Conte-Burica.

### Ethnobotanical characteristics of most salient *krigä kwedäga*

**Fig. 7:**
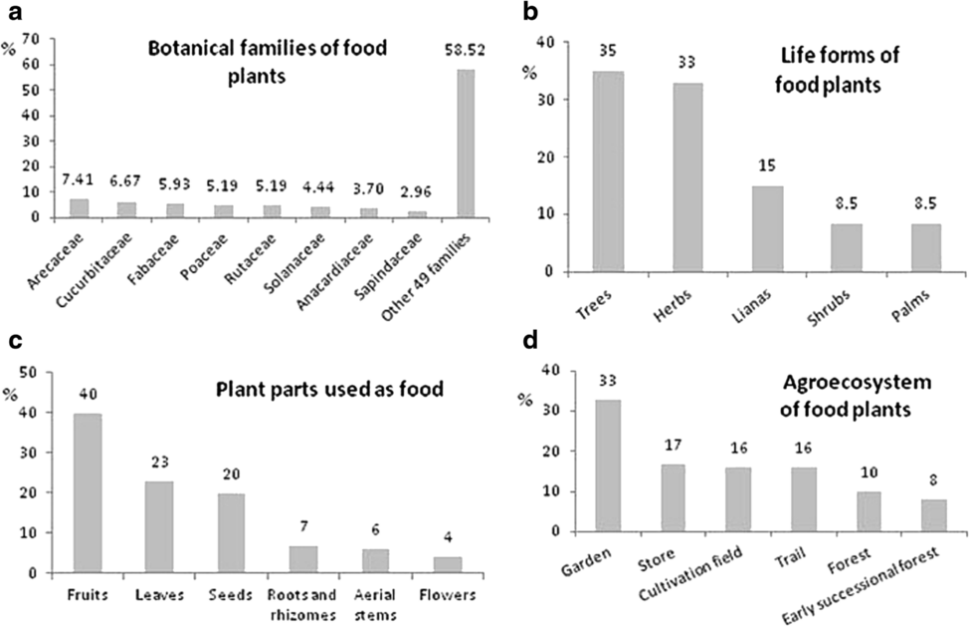
Ethnobotanical characteristics of 135 identified plants within the cultural domain of food plants. **a**) Botanical families; **b**) life forms; **c**) plant parts used; **d**) agroecosystem moslty found (includes stores)

Subsequent results on the overall culinary repertoire are based on the identified plants to genus or species level, that is 135 taxa (96.4 % of the domain), whilst excluding unidentified plants (5 different taxa) (See Appendix 2). The botanical spectrum of food plants found in Southern Conte-Burica during this study includes 57 botanical families and 110 genera (Fig. 7). Nine botanical families have four or more edible species in Conte-Burica: Arecaceae being the most significant one (10 spp. in 9 genera), followed by Cucurbitaceae (9 spp. in 5 genera), Fabaceae (8 spp. in 7 genera), Poaceae (7 spp. in 7 genera), Rutaceae (7 spp. in one genus), Solanaceae (with 6 spp. in 4 genera), Anacardiaceae (5 spp. in 3 genera), and Sapindaceae (with 4 spp. in 4 genera). These plants were reportedly found predominantly in cultivated areas (33 % in homegardens and 16 % in cultivation fields), but a third were found in forested (10 %) or less managed agroecosystems (trails with 8 % and successional forests with 8 %), and roughly 17 % were obtained in shops. Nevertheless for most families, and depending on the season, basic ingredients are now provisioned from store foods, with only about a fifth deriving from cultivated foods and another fifth from less managed ecosystems.

Taking into account only the 25 key food plants obtained from cultural domain analysis, ethnobotanical and agroculinary characteristics regarding lifeform, organs used, location in agroecosystem, seasonality and type of food were explored in focused interviews with adult key informants and in structured interviews amongst primary informants (Table 3). Table 3 summarizes vernacular and botanical names, taxonomic family, life forms, plant organ used, agroecosystem mostly found, and freelisting results (frequency, average rank, and Smith's salience) with percentage values of overall ethnobotanical characteristics for key food plants.

**Table 3:** Key food plants in Southern Conte-Burica, Costa Rica. Vernacular and botanical names, botanical family, life form, plant organ used, agroecosystem mostly found, and freelist results (frequency, average rank and Smith's salience) for key food plants, in decreasing Smith’s saliency order. (*n* = 10 key informants; key plants = 25 folk taxa)

Ngäbere Name	English Name	Spanish Name	Scientific Name	Botanical Family	Frequency (%)	Average Rank	Smith's Salience (S)	Life form^a^	Plant organ used^b^	Agroecosystem mostly found^c^
Aro	Rice	Arroz	Oryza sativa	Poaceae	100	6.20	0.847	H	S	CuA, To
Mumá	Bean (common)	Frijol (común o cañero), vainica	Phaseolus vulgaris	Fabaceae (Papil.)	100	7.40	0.822	L	S, Frt	CuA, To
Mumá krire	Pigeon pea	Frijol de palo	Cajanus cajan	Fabaceae (Papil.)	100	8.20	0.801	S	S, Lt	CuA
I	Corn	Maíz	Zea mays	Poaceae	90	10.44	0.664	H	S	CuA, *To*
Ö	Manioc, cassava	Yuca	Manihot esculenta	Euphorbiaceae	90	13.78	0.645	H	R, Lt	CuA, *Ga*
Naran	Orange	Naranja	Citrus × sinensis	Rutaceae	90	14.33	0.552	T	Fr	Ga, *Tr*
Tä	Arrowleaf, otoe, yautia	Tiquizque (chamol, otoe)	Xanthosoma spp.	Araceae	80	16.50	0.538	H	R, Lt	CuA, *Ga*
Drun	Yam	Ñame	Dioscorea alata	Dioscoreaceae	100	21.20	0.497	L	R	Ga, Cu
Baran bun	Plantain	Plátano	Musa acuminata × balbisiana	Musaceae	80	15.13	0.465	H	Fr, L (I), (T)	Ga, CuP
Kä	Cocoa	Cacao	Theobroma cacao	Sterculiaceae	70	15.00	0.458	T	S, S (A)	Ga, CuP
Mumá köire	Cowpea	Frijol de bejuco, caupí	Vigna unguiculata	Fabaceae (Papil.)	60	10.33	0.449	L	S	CuA
Kabe	Coffee	Café	Coffea arabica	Rubiaceae	70	14.57	0.447	S	S	To, *Ga*, *CuP*
Barän rugá	Banana	Banano	Musa acuminata	Musaceae	90	19.56	0.442	H	Fr, L (I), (T)	Ga, CuP
Drune	Yampi	Ñampí	Dioscorea trifida	Dioscoreaceae	90	21.78	0.429	L	R	Ga, Cu, *Tr*
Bé	Pumpkin, Squash	Ayote (auyama)	Cucurbita moschata	Cucurbitaceae	70	19.86	0.376	L	Lt, Fr	Ga, Cu
Muá	Pineapple	Piña	Ananas comosus	Bromeliaceae	70	19.71	0.367	H	Fr	Ga, Cu
Jlimu	Lemon	Limón criollo	Citrus × limon	Rutaceae	60	16.33	0.351	T	Fr	Ga
Dugá	Avocado	Aguacate	Persea americana	Lauraceae	50	11.20	0.339	T	Fr	Ga, Tr, ESF
Söran	Soursop	Guanábana	Annona muricata	Annonaceae	60	19.50	0.315	T	Fr	Ga
Mölen	Heliconia	Chichica	Heliconia spp.	Heliconiaceae	80	22.38	0.303	H	Lt, Flt, Tt	Ga, Sh, ESF, Cu
Mraño	Cashew	Marañón	Anacardium occidentale	Anacardiaceae	50	19.20	0.277	T	Fr, S, L (I)	Tr, Ga
Dabá	Peach palm	Pejibaye	Bactris gasipaes	Arecaceae	60	23.50	0.26	P	Fr, Lt	CuP, Fo
Mitdrá	Several palms	Palmito de palma real/corozo	Attalea butyracea, Bactris gasipaes and other minor species	Arecaceae	50	21.40	0.216	P	Lt, Fr	Tr, ESF, Fo
Kolan	Wild coriander	Culantro (coyote)	Eryngium foetidum	Apiaceae	90	31.22	0.198	H	C (L)	Ga
Bere	Breadnut	Berbá, ojoche, lechoso	Brosimum alicastrum	Moraceae	50	24.80	0.175	T	Fr, S	Fo
			Percentages							37.5 % Cu
				12 % Arecaceae					34 % Fr	33.5 % Ga
				12 % Fabaceae				40 % H	24 % S	8 % Tr
				8 % Dioscoreaceae				28 % T	21 % L	6.8 % Fo
				8 % Musaceae				16 % L	10 % R	6.8 % ESF
				8 % Poaceae				8 % P	8 % T	5.1 % To
				8 % Rutaceae						
				44 % in 11 other fam.				8 % S	3 % Fl	2 % Sh

Abbreviations: ^a^ *Life form*: T: Tree; S: Shrub; H: Herb; L: Liana, vine; P: Palm; ^b^ *Plant organ used*: R: Storing organs (Roots, rhizomes, tubers, and bulbs), usually underground; L: Leaves; Fl: Flowers; T: Stems (includes bark); t: tender (several parts); Fr: Fruits (exo- and/or mesocarp); S: Seeds; A: Arillus; C: Condiments; Ed: Sweetener; Ac: Oil; Col: Food colouring; I: Indirect uses; ^c^ *Agroecosystem mostly found*: Ga: Garden; CuA: Annual cultivation field (beans, corn- broadcasting- & rice, yuca, tiq.- slash and burn); CuP: Pluriannual cultivation field (cocoa, coffee, B/P, peach palm, cane); Sh: Shrubland; ESF: Early succesional forest; Fo: Forest; Ri: River; We: Wetland; Be: Beach; Tr: Trails; To: Town (& store). Parenthesis in plant part used and italics in agroecosystems indicate minor significance. Final note: One plant may have several plant organ used and agroecosystem mostly found (See Appendix 2 for a full list of food plants recorded with all participants)

**Fig. 8:**
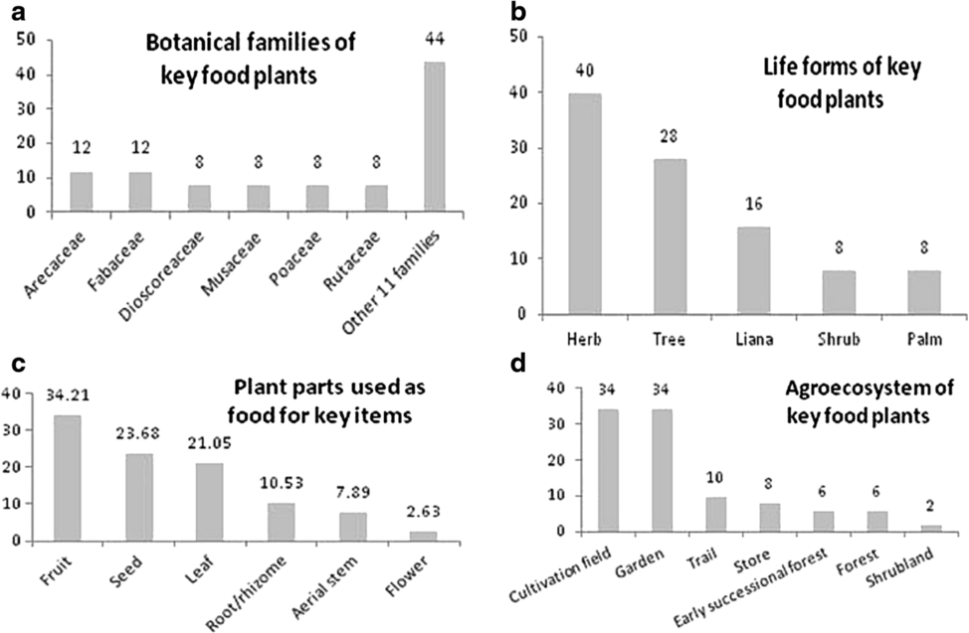
Ethnobotanical characteristics of 25 key food plants. **a**) Botanical families; **b**) life forms; **c**) plant parts used; **d**) agroecosystem moslty found (includes stores)

These 25 key food plants (Fig. 8) are from 17 different botanical families (56 % dicots and 44 % monocots). According to seasonality of key food plants (figures not shown), 44 % of edibles can be obtained annually, 40 % seasonally and 16 % sporadically. With regard to geographical origin, 64 % have an American origin (28 % Tropical America, 20 % South America, 16 % Mesoamerica), 28 % originated in South East Asia, and 8 % in Africa (4 % Western Africa, 4 % Ethiopia). Fifty culinary preparations were recorded for key food plants, indicating an average of 2 distinct preparations per plant. Most food plants are consumed boiled (including in soups) (30 % of culinary preparations) or eaten raw (usually as snacks) (20 %). Other preparations included alcoholic preparations (*chicha*) (10 %), natural juices (*fresco*) (8 %), and coffee or coffee substitutes (6 %), roasted and barbequed foods (6 %), and fried foods (4 %). Porridges, tamales, puff pastries, *tortillas*, raw in salads, marinated in lemon (*ceviche*), chopped (*picadillo)*, and in omelets were the most infrequent means of preparation at 2 % each. Amongst key food species, *chicha* was mentioned to be prepared from the following: maize (*Zea mays*), cassava (*Manihot esculenta*), plantain and banana (*Musa* spp.), pineapple (*muá*, *Ananas comosus*) (sometimes including *Oryza sativa*), peach palm (*dabá*, *Bactris gasipaes*) and breadnut (*bere*, *Brosimum alicastrum*). In relation to major nutritional characteristics of key food plants, most botanical elements correspond to ingredients rich in vitamins, minerals and fiber (40 %, usually juicy fruits and leaves), followed by foods rich in carbohydrates (36 %, usually grains, starchy fruits and roots), proteinaceous foods (16 %, usually pulses) and fatty foods (8 %).

### Perceived change in local production and dietary provisioning of key food plants

**Fig. 9:**
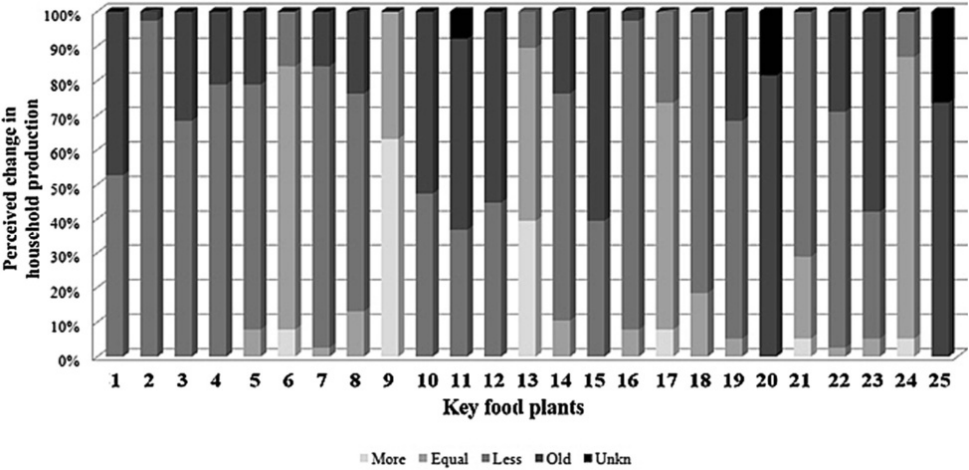
Perception of change in household production of key food plants (in descending Smith's salience order). (*n* = 38 informants). Items: 1 = Aro (*Oryza sativa*); 2 = Mumá (*Phaseolus vulgaris*); 3 = Mumá krire (*Cajanus cajan*); 4 = I (*Zea mays*); 5 = Ö (*Manihot esculenta*); 6 = Naran (*Citrus × sinensis*); 7 = Tä (*Xanthosoma* spp.); 8 = Drun (*Dioscorea alata*); 9 = Baran bun (*Musa acuminata × balbisiana*); 10 = Kä (*Theobroma cacao*); 11 = Mumá köire (*Vigna unguiculata*); 12 = Kabe (*Coffea arabica*); 13 = Rugá (*Musa acuminata*); 14 = Drune (*Dioscorea trifida*); 15 = Bé (*Cucurbita moschata*); 16 = Muá (*Ananas comosus*); 17 = Jlimú (*Citrus × limón*); 18 = Dugá (*Persea americana*); 19 = Söran (*Annona muricata*); 20 = Mölen (*Heliconia* spp.); 21 = Mraño (*Anacardium occidentale*); 22 = Dabá (*Bactris gasipaes*); 23 = Mitdrá (*Attalea butyracea* and *Bactris gasipaes*); 24 = Kolan (*Eryingium foetidum*); 25 = Bere (*Brosimum alicastrum*)

Perceived change amongst key food plants was explored with primary informants (*n* = 38) using structured interviews, including a rating exercise for each of the 25 key species using a five point scale, as described in the Methods section. Results are presented and summarized in Fig. 9. Linked to these results on perceived change, an article with a detailed analysis of the explanations given by the Ngäbe in Conte-Burica for the agricultural and culinary changes detected is under preparation.

The previous figure shows how key species are perceived to have changed regarding local household production in a heterogeneous sample of 38 informants. While most taxa are perceived to have decreased (‘less’ or reduced category) or have completely disappeared in household production (‘old’ or abandoned category) such as rice, beans, maize, cassava, arrowroot, yam and several perennial crops, only a few are said to have been maintained or increased their local production, such as *Musa* spp. and *Citrus* spp. Those species that include a higher frequency of ‘unknown’ responses, such as *Heliconia* spp. and breadnut tree, are in fact non-staple species that are being lost—some younger participants no longer realize they are edible. Diagrams such as in Fig. 9, give a generalized idea about changes in local production or provisioning of different plants (or kinds of agroecosystems) at different times, as well as intracultural variation in the perception of change (per individual, household or hamlet).

## Discussion

### Ngäbe culinary repertoires

Results show a domain of food plants composed of 140 species of which 85 % are still available in the area. The 25 most salient taxa include a mixed repertoire of introduced crops (*Oryza sativa*, *Cajanus cajan*, *Citrus* spp., *Musa* spp.) with local plants (*Phaseolus vulgaris*, *Zea mays*, *Manihot esculenta, Xanthosoma* spp*.*); while macronutritionally these indicate a preponderance of starches and proteins. Interestingly, the domain contains significant local crops that were once dominant in the diet during Pre-Columbian times, such as *Cucurbita moschata*, *Ananas comosus*, *Persea americana*, *Annona muricata* amongst several others [7]. As already pointed out by Castañeda and Stepp [16], plantains and bananas still play an important role in Ngäbe diets with a high number of available varieties, and are thus accorded high saliency in freelists. Thus, the domain of food plants shows both evidence of dietary transition and the maintenance of heritage foods.

On the other hand, the domain does not capture some important changes taking place in local foodways, especially when it comes to changes in the sources and processing of important foods. Thus while rice, coffee, beans and plantain-bananas constitute the most consumed food plants in Southern Conte-Burica today, industrial palm oil, industrial wheat and refined sugar should be added to these as they represent key ingredients in Southern Conte-Burican diets. However, these latter three major ingredients were not mentioned by informants in freelists. A similar phenomenon occurs with coffee, which although a dominant item in the diet, has a rather low saliency compared to its frequency of use. Despite their origins in plants, these food items are seemingly no longer associated with food plants, and instead are considered as “foods”, perhaps because they are processed and now bought in stores. In fact, most consumed food and beverage staples, such as rice and coffee, are bought in stores today, while most accompanying foods, such as beans, yams and manioc, and snacks, such as fruits, are derived from local sources. The increasing disconnect between food items and their origins as plants is one indicator of a loss of local ethnobotanical knowledge accompanying Ngäbe foodways transitions.

### Local perceptions of agricultural and ethnobotanical change

As results on perceived change indicate (Fig. 9), many key food plants are seen by the Ngäbe to have decreased considerably in local production, rice and beans being the most significant, while others are considered no longer used, such as *Heliconia* spp. (*mölen*) and *Brosimum alicastrum*. Among the youth several of these “older” food plants are unknown (such as *Vigna* beans, *Heliconia* spp. and *Brosimum alicastrum*), most likely because they have never consumed them. In a region commonly known for its subsistence agricultural production [3, 38], a detailed agroculinary analysis indicates that today, on average, local production contributes to self-sufficient consumption in only a very few families, and in most cases it only does so sporadically for non-staple foods. It has almost disappeared in several households and especially in relation to highly consumed foods such as rice; these are striking facts that might have been overlooked without a detailed agroculinary analysis of change [2].

Underlying these changes in food consumption, then, are changes in agricultural production and more generally, household livelihood systems. Ngäbe readily admit that as economic opportunities continue to expand, and there does appear to be a trend toward diversification of economic activities at the household level, then available labor for any one activity becomes stretched. It is this trend primarily (but see discussion below) that Ngäbe recognize as leading to the abandonment of more labor intensive agricultural crops. Annually cultivated species in decline include rice—which had been both the desired staple and the main cash crop—beans, maize, and non-herbaceous homegarden and perennial crops. Crops that require higher labor inputs, such as rice or maize, are reported to have been abandoned first. In some cases, however, families stop producing specific crops for several years and then, when a need arises, start producing them again. As Fig. 9 shows, the exception to the general trend is in *Musa* and *Citrus* varieties, which are believed to be expanding because these have never been commercially sold nor bought, so people depend on their gardens and orchards to provide them. They are also seen as preferred foods and are available year round, which drives their cultivation and the proliferation of many land races. In terms of labor inputs, being perennials that either reproduce vegetatively (*Musa* spp*.*) or are easy to propagate and live long, they require less labor to harvest and maintain and therefore “fit” into a newly developing livelihood strategy that emphasizes less agricultural production. It appears, therefore, that Ngäbe understanding of change supports the basic *nutritional transition* hypothesis described in the introduction. However, related and synergistic transformations in land use, biocultural diversity and state policies, add complexity, as well as some positive consequences, to the Ngäbe explanation of change in their foodways, thereby nuancing the nutritional transition model.

One positive change is that as a result of the decrease in crop production, the amount of land under swidden cultivation has decreased, and forest cover is said by the Ngäbe to be expanding. For many generations, shifting or swidden cultivation was the vital agricultural system of the Ngäbe [10], and other peoples living in the tropical forests of Latin America [53]. The recent decline of its prevalence across Latin America is in many places driven by the permanent conversion of forests to intensive agriculture and ranching, supported and subsidized by government policies. As Fox et al. conclude: "swiddening has always been characterized by change, but since the onset of modern independent nation states, government policy and the expansion of capitalism in new forms have transformed the landscape and swidden practices through mechanisms that are different in the extent and depth of their landscape effects than ever before" [54]. In the Ngäbe territories, however, the same pro-development processes are only partly responsible for some transformations of the forest landscape. While plantations appear to have come and gone in the area, new economic activities and new government programs in education and conservation are driving an expanding market economy that is in fact causing the suspension of swidden farming for key staple crops and an increase in the extent of older fallow forests, as recognized by a number of participants while analyzing causes and consequences of agricultural and dietary transitions.

While expanding forest cover may be good for biodiversity, and the Ngäbe are aware of this benefit, the decline in the diversity of food stuffs, and the agricultural and other food provisioning and preparation practices that support it, means that biocultural diversity is in fact under pressure. The abandonment of agriculture and other provisioning activities by the younger generation reduces the formation of agricultural experiences, agricultural and ecological knowledge of the land, the climate and weather, the variety of harvested and available plants, symbolic elements or the tools used, amongst many other matters relevant to ethnobiologists [55–57]. The same happens with transformations in post-harvest and transactional activities (i.e., exchange and distribution), cooking methods, and their associated knowledge-practice-belief systems. In addition, the uniqueness of Conte-Burica’s 'natural resources market' must be noted, especially in an area where it is still so abundant. Without neglecting the positive effects of entering a mixed economy, such as diminishing the need for underpaid wage labor―as experienced by many Ngäbe in the past―the accelerated reduction of local self-sufficiency in Conte-Burica cannot be left aside. The transformation in foodways processes changes the configuration of agroculinary experiences, knowledge, methods, tools, practices, attitudes and beliefs, which clearly alter previously existing human-plant interactions in the area. In addition, specific foodways of certain foods undergo major transformations, in some cases being completely lost while others appear anew or acquire new uses.

As mentioned above, government policies in education and conservation are also recognized by Ngäbe as contributing to these transformations of their agroculinary systems. For several years now, parents of children have been receiving grants for every child that attends formal government schools. This leads to the disruption of previous non-formal learning processes and institutions that promoted the transmission of agroculinary knowledge; it also leads to greater incorporation in the market economy as education subsidies allow purchasing of basic staple foods. Added to this is the introduction of a *Payment for Ecosystem Services* program funded by Governmental agencies, which encourages forest conservation and protection, but also further endows families with the means to subsist without agriculture. Thus both development and conservation programs are accelerating acculturation to a market based economy and dependence on store bought food stuffs, at the expense of agroculinary systems and biocultural diversity, and with clear consequences for health and wellbeing. Such a profound transformation supports calls by ethnobiologists and others for in-depth investigations of the complexity and dynamics of nutritional transformations and their relationships to ethnobotanical knowledge, biocultural diversity, health and wellbeing [58–60].

Hence, when backed by ethnobiological observations and emic explanations (D’Ambrosio and Puri, in preparation), the data make a compelling argument against the position that sustainable development initiatives such as PES and conditional cash transfers per se protect biocultural diversity, alleviate poverty and improve rural or marginalized populations. In the Conteburican case, conservation and development programs—based concurrently on social democrat and neoliberal paradigms—may have had the opposite effect in certain livelihood domains or for certain individuals and households. For instance, certain crops, agroecosystems, recipes, knowledge, practices, and other biocultural expressions linked to food seem to be disappearing as younger generations disengage from 'older ways', while food autarky and self-sufficiency has been greatly affected, even with the increasing existence of governmental and non-governmental programs and funding.

Decontextualized formal schooling programs, large amounts of money centralized by a few locals, and personal choices (conscious and unconscious), also contribute as accelerators of acculturation processes. Such initiatives also affect internal community integrity and power relationships, while casting doubt on the assumptions that there exists a vast grassroots commitment to community within indigenous societies, or that they hold a unique and distinctive connection with nature, as the great variability amongst individuals and households has shown. Both respondents’ and our own observations suggest that while formal education may be an important driver in promoting an “extinction of experience” (sensu Nabhan and St Antoine [61]), with regard to food plants, of greater significance is expanding economic diversification and reduced availability of labor, coupled with increased government subsidies for education and conservation, which is driving agroculinary transformations that lead to knowledge erosion and loss of biocultural diversity [60, 62–65].

## Conclusions

In this paper, we have introduced the results of ethnobotanical research associated with culinary traditions, documenting the rather vast knowledge of food plants and their perceived changes by a significant sample of the Ngäbe population in Conte Burica. We have concluded that while consensus exists within the domain, variations in local production and consumption significantly influence the erosion of knowledge of food plants and their associated agroculinary practices and beliefs, especially among Ngäbe youth. Interestingly, environmental degradation is not necessarily part of this story, with the environmental costs of greater involvement in the market economy being displaced outside the Ngäbe territories.

This study plays, we believe, a significant role in supplementing the limited pool of current ethnobotanical literature on nutritional transitions through documenting and understanding how Conte Burica’s “post-traditional” Ngäbe food systems evolve and politically transform and develop. The study intends to contribute to a deeper understanding of transformations through documenting the changing ethnobotanical and culinary systems surrounding the Ngäbe’s most esteemed food plants, a research subject which is virtually non-existent for Central and South American social-ecological systems. Importantly, post-traditional foodways analysis is ideally suited to raising public understanding of the significance of political-ecology/economy and historical ecology approaches to the study of diversity and complexity in the agricultural, horticultural, and ethnobiological contexts.

### Ethics approval and consent to participate

Prior informed consent was obtained verbally before interviews and other activities were conducted, with the aims, methodology, and outcomes of the study being explained to all participants. We followed ethical guidelines adopted by the International Society of Ethnobiology (2006) and research was evaluated and approved by the Ethics Committee of the School of Anthropology and Conservation at the University of Kent at Canterbury, UK.

### Consent for publication

Not applicable.

## Figures and Tables

**Table 5:**
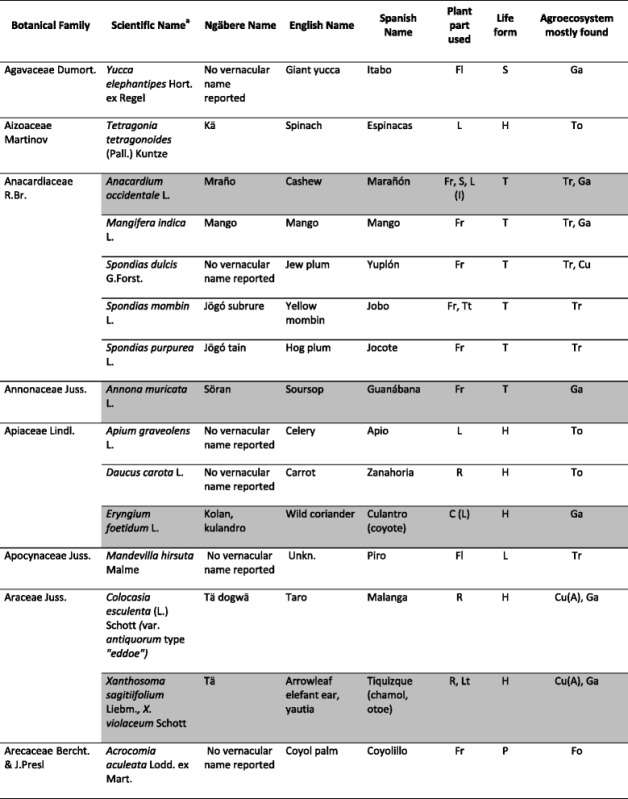
Cultural domain of food plants (140 items) including plants elicited in freelists, as well as those derived through interviews and inventories. (*n* = 72 informants)

*Abbreviations*: *Plant part used*- R: Storing organs (Roots, rhizomes, tubers, and bulbs), usually underground; L: Leaves; Fl: Flowers; T: Stems (includes bark); t: tender (several parts); Fr: Fruits (exo- and/or mesocarp); S: Seeds; A: Arillus; C: Condiments; Ed: Sweetener; Ac: Oil; Col: Food colouring; I: Indirect uses; *Life form*- T: Tree; S: Shrub; H: Herb; L: Liana; P: Palm; *Agroecosystem mostly found*- Ga: Garden; CuA: Annual cultivation field (beans, corn- broadcasting- & rice, cassava, yautia.- slash and burn); CuP: Pluriannual cultivation field (cocoa, coffee, banana-plantain, peach palm, sugarcane); Sh: Shrubland; ESF: Early succesional forest; Fo: Forest; Ri: River; We: Wetland; Be: Beach; Tr: Trails; To: Town (& store). Unkn.: Unknown

Angiosperms (identified)- In gray, key species derived from cultural domain analysis

^a^The International Plant Names Index (2012). Published on the Internet http://www.ipni.org [accessed 26 January 2013]

## Appendix 1

**Table 4:** List of 102 ethnotaxa derived from freelisting elicitations (in decreasing Smith’s salience index), constituting the initial cultural domain of food plants in Conte-Burica. (*n* = 10 key informants)

Number	Ngäbere name	Spanish name	Smith's Salience	Frequency	Average rank
1	Aro	Arroz	0.847	100	6.2
2	Mumá	Frijol cañero (común)	0.822	100	7.4
3	Mumá krire	Frijol de palo	0.801	100	8.2
4	Ï	Maíz	0.664	90	10.44
5	Ö	Yuca	0.645	90	13.78
6	Naran	Naranja	0.552	90	14.33
7	Tä	Tiquizque	0.538	80	16.5
8	Drun	Ñame	0.497	100	21.2
9^b^	Baran bun	Plátano	0.465	80	15.13
10	Kä	Cacao	0.458	70	15
11	Mumá köire	Frijol de bejuco	0.449	60	10.33
12	Kabe	Café	0.447	70	14.57
13^b^	Baran rugá	Banano	0.442	90	19.56
14	Drune	Ñampí	0.429	90	21.78
15	Bé	Ayote	0.376	70	19.86
16	Muá	Piña	0.367	70	19.71
17	Jlimu	Limón criollo	0.351	60	16.33
18	Dugá	Aguacate	0.339	50	11.2
19	Söran	Guanábana	0.315	60	19.5
20	Mölen	Chichica	0.303	80	22.38
21	NVNR	Mango	0.296	40	11.5
22	NVNR	Mandarina	0.292	40	14
23	Tä	Chamol	0.281	50	21.6
24	Mraño	Marañón	0.277	50	19.2
25	Kegemá	Papaya	0.27	50	22.8
26	Jlimu malen	Limón dulce	0.269	40	16.25
27	Dabá	Pejibaye	0.26	60	23.5
28	Ñajú	Ñajú	0.254	40	14.25
29	Jlimu kwaga	Limón agrio	0.238	40	20.25
30	Kogo	Coco (pipa)	0.232	40	20.5
31	Bü	Guaba	0.231	40	19.75
32	Mi	Camote (batata)	0.228	30	11.67
33	Mumá kedebá	Habas	0.219	30	11
34	Mitdrá	Palmito	0.216	50	21.4
35	Sabo	Zapote	0.208	40	18.25
36	Kuguoli	Zorrillo.	0.201	50	27.2
37	Kolan	Culantro coyote	0.198	90	31.22
38	Mumá poroto	Frijol poroto	0.194	30	14
39	Nibá malen	Chile (dulce)	0.187	50	30.6
40	Bere	Berbá, ojoche	0.175	50	24.8
41	NVNR	Mamón	0.174	30	21.33
42	Tä dogwä	Malanga	0.173	40	25.75
43	Ibia malen	Caña de azúcar	0.166	30	18.33
44	NVNR	Verdolaga	0.147	40	30.5
45	Ï tain	Maíz pujagua	0.14	20	11
46	NVNR	Manzana de agua	0.14	30	26.33
47	Gwate	Maracuyá	0.139	30	25
48^a^	Dö	Chicha	0.133	30	19.67
49	Be	Chayote	0.132	20	18
50	NVNR	Carambola	0.129	30	28.67
52	NVNR	Ajo	0.126	40	31.25
51^b^	Cuadale	Cuadrado	0.126	50	28
53	NVNR	Pipián	0.119	20	21
54	NVNR	Bambú tierno	0.117	20	19.5
55	Bodá	Chidra (chira)	0.112	20	21
56	NVNR	Itabo	0.103	50	39
58	Migá	Nance	0.102	20	16
57	Söga	Jaboncillo	0.102	20	25
59	NVNR	Hierba (bejuco) de ajo	0.1	50	33.2
60	Toare	Tomate	0.099	20	24.5
61	NVNR	Naranjilla	0.097	20	25
62	NVNR	Melón	0.088	20	27
63	NVNR	Camote de monte	0.082	10	9
64	NVNR	Sandía	0.081	20	31
66	Nimá	Guayaba	0.076	10	13
65	NVNR	Noni	0.076	20	29
67	NVNR	Cebolla	0.074	20	24
68	Nomón	Mamey	0.073	10	13
69	Nomó krie	Níspero de montaña	0.07	10	14
70	NVNR	Almendro	0.066	10	16
72	Mumá Carnita	Carnita (frijol)	0.063	10	19
71	Kiongodä	Helecho	0.063	30	38.67
73	NVNR	Orégano carnoso	0.06	40	36.25
74	Chari	Toronja	0.059	10	22
75	Kimó	Estococa	0.057	30	36.67
76	Ñürun	Pacaya	0.054	30	40.67
78^b^	Tendemani	Banano manzana	0.051	20	32.5
79^a^	Miá	Chicheme	0.051	10	26
77	NVNR	Piro	0.051	20	31
81	Suligrie	Mamón chino	0.049	20	22.5
80	Pimienta	Pimienta	0.049	10	26
82	cf. Kadoguä	Ñampí silvestre	0.045	10	18
84	Beregrie	Castaño	0.042	10	16
85	NVNR	Camote de monte	0.042	10	19
83	NVNR	Zanahoria	0.042	10	16
86	NVNR	Avena	0.037	10	32
87	Ragä	Chayotillo	0.035	10	21
90	NVNR	Canela	0.027	10	37
88	Mölögwä	Palma real	0.027	20	41.5
89	Ngomó	Zapotillo	0.027	10	20
92	Klisebá	NVNR	0.024	20	25.5
91	NVNR	Sucanca	0.024	20	43
93	NVNR	Coliflor	0.023	10	21
94	Bugrüm	Ortiga	0.022	20	34
95	Sa krie	Garrobo	0.019	10	26
96	NVNR	Repollo	0.019	10	22
99	Kwaga kriblu nué	Bejuco de flor blanca	0.012	10	24
98	Nibá dime	Chile picante	0.012	10	46
97^b^	Bechi, muachi	Guineo	0.012	20	36.5
100	Kurä	Achiote	0.011	10	40
101	NVNR	Orégano (de la tienda)	0.004	10	44
102^b^	Pelipita	Felipita	0.002	10	45

NVNR: No vernacular name recorded

^a^: Items that do not correspond to a specific plant, but type of preparation made with several plants from the list

^b^: Six different kinds of *Musa* A × A and *Musa* A × B

## Appendix 2

**Table 6:** Angiosperms (unidentified)

Botanical Family	Scientific Name	Ngäbere Name	English Name	Spanish Name	Plant part used	Vital form	Agroecosystem mostly found
Unknown	Unknown	Unknown	Unknown	Sucanca	Fl	P	Fo
Unknown	Unknown	Klisebá	Unknown	Unknown	L, Fl	L	Fo
Unknown	Unknown	Ragä	Unknown	Chayotillo	Lt, Tt	L	CuA
Unknown	Unknown	Kadogwä?	Unknown	Ñampí silvestre	R	L	Fo
Unknown	Unknown	Unknown	Unknown	Camote de monte	R	L	Fo

**Table 7:** Ferns (Pteridophyta)

Botanical Family	Scientific Name	Ngäbere Name	English Name	Spanish Name	Plant part used	Agroecosystem mostly found
Dennstaedtiaceae Stace	*Hypolepis repens* (L.) C.Presl	Kiongodä	Fern	Helecho	L (Frond)	Sh, ESF, Fo

**Table 8:** Fungi (Ascomycetes)

Botanical Family	Scientific Name	Ngäbere Name	English Name	Spanish Name	Plant part used	Agroecosystem mostly found
Sarcoscyphaceae (Order Pezizales)	*Cookeina* spp. Kuntze	Kri olo	Ear mushroom	Hongo oreja	Fruiting body	Fo, ESF

The current work follows the latest *Manual de plantas de Costa Rica* [68–72], with monocotyledonous families adhering to Dahlgren et al. [73], while dicotyledonous families follow Cronquist [74]

## References

[1] Borge C, Castillo R (1997). Cultura y Conservación en la Talamanca Indígena.

[2] D’Ambrosio U (2013). Ngäbe agroculinary transitions in Costa Rica.

[3] Camacho C (1996). En la frontera del siglo XX.

[4] Instituto Nacional de Estadística y Censos-Costa Rica (INEC). Preliminary data from the 2011 census on indigenous territories. C 16. Costa Rica Población indígena por pertenencia a un pueblo indígena, según provincia y sexo. 2012. Available at: http://www.inec.go.cr. [Accessed 15 August 2012].

[5] Constenla A (2011). La diversidad lingüística de Costa Rica: Las lenguas indígenas. Filología y Lingüística.

[6] Solano E. La población indígena en Costa Rica según el censo 2000. In: Costa Rica a la luz del Censo del 2000. Centro Centroamericano de Población (CCP) - Proyecto Estado de la Nación. San José, Costa Rica: Instituto Nacional de Estadísticas y Censos (INEC); 2004. 341–73.

[7] Staller JE, Carrasco MD, Staller JE, Carrasco MD (2010). Pre-Columbian foodways in Mesoamerica. Pre-Columbian Foodways: Interdisciplinary Approaches to Food, Culture, and Markets in Ancient Mesoamerica.

[8] Pérez H (2005). La dinámica demográfica de las poblaciones indígenas del trópico húmedo en América Central (censos 2000).

[9] Constenla A (2008). Estado actual de la subclasificación de las lenguas chibchenses y de la reconstrucción fonológica y gramatical del protochibchense. Estudios de Lingüística Chibcha.

[10] Young PD (1971). Ngawbe: Tradition and Change among the Western Guaymí of Panama. Illinois Studies in Anthropology, no. 7.

[11] Karkotis A (2011). Now we live together.

[12] Camacho-Zamora JA (1983). Etnobotanica Cabecar. America Indigena.

[13] Hazlett DL (1986). Ethnobotanical observations from Cabecar and Guaymí settlements in Central America. Econ Bot.

[14] Madriz JP (1999). Explotación etnobotánica en los bosques húmedos tropicales de la Reserva Indígena Tayní, Costa Rica. Revista Forestal Centroamericana.

[15] Zaldivar ME, Rocha OJ, Castro E, Barrantes R (2002). Species diversity of edible plants grown in homegardens of Chibchan Amerindians from Costa Rica. Hum Ecol.

[16] Castaneda H, Stepp JR (2007). Ethnoecological Importance Value (EIV) Methodology: Assessing the Cultural Importance of Ecosystems as Sources of Useful Plants for the Guaymi People of Costa Rica. Ethnobot Res Appl.

[17] Castañeda H (2004). Ethnobotanical analysis of different successional stages as sources of wild edible plants for the Guaymi people in Costa Rica.

[18] Koshear J (1995). Guaymi agriculture, forest utilization and ethnobotany in Coto Brus.

[19] Gordon BL (1982). A Panama forest and shore. Natural history and American culture in Bocas del Toro.

[20] Joly LG, Guerra S, Septimo R, Solis M, Correa M, Gupta M (1987). Ethnobotanical inventory of medicinal plants used by the Guaymi Indians in Western Panama. Part 1. J Ethnopharmacol.

[21] Joly LG, Guerra S, Septimo R, Solis M, Correa M, Gupta M (1990). Ethnobotanical inventory of medicinal plants used by the Guaymi Indians in Western Panama, Part II. J Ethnopharmacol.

[22] Bletzer KV. Social origins of folk illness among Ngawbere of the northern Valiente peninsula: The case of “chakore” and “ha ko botika”. East Lansing: Michigan State University Doctoral thesis; 1988.

[23] Border H (2011). Ethnobotany of the Ngäbe People of Panama.

[24] Pastrana A (1998). El componente arbóreo en los sistemas agroforestales tradicionales: prioridades y potencialidades de los indígenas Ngöbe. "La Gloria", Changuinola-Panama. Master's thesis.

[25] Pastrana A, Lok R, Ibrahim M y E. Víquez. El componente arbóreo en sistemas agroforestales tradicionales de los indígenas Ngöbe, La Gloria, Changuinola, Panama. Revista Agroforestería en las Américas. 1999;6(23):69–71.

[26] Méndez E, Calvo G, Ortiz M (1999). Caracterizacion de la comunidad Ngobe de Valle de Risco, Bocas del Toro, Panama (Characterization of the Ngobe community of Valle de Risco, Bocas del Toro, Panama). Revista Forestal Centroamericana (Costa Rica).

[27] Montero F, Corrales J (2008). Hojas, flores y tallos comestibles no tradicionales en Costa Rica. Rev. Ciencias Sociales.

[28] Carbonell F (1998). Uso y abundancia de fauna en una comunidad indígena Ngäbe (Guaymí) en Punta Burica y su relación con la conservación en costa rica. Master's thesis.

[29] Altrichter M, Carbonell F, Tavares R, D’Ambrosio U (1999). Diagnose and evaluation study (field research phase) for the project “Community participation in training processes and elaboration of educational materials on wildlife conservation in Costa Rica” (in Spanish).

[30] Organization for Tropical Studies. Undergraduate opportunities. Student articles and course books. 2015. Available at: http://education.tropicalstudies.org/en/education/undergraduate-opportunities/student-articles-and-course-books.html. [Accessed 10 November 2015].

[31] Ngäbe’s Traditional Doctors’ Council (2001). Krägä täräe Ngäbere. Indigenous Territories of Costa Rica.

[32] Popkin BM, Horton S, Kim S, Mahal A, Shuigao J (2001). Trends in diet, nutritional status, and diet-related noncommunicable diseases in China and India: the economic costs of the nutrition transition. Nutr Rev.

[33] Uauy R, Albala C, Kain J (2001). Obesity trends in Latin America: transiting from under- to overweight. J Nutr.

[34] Albala C, Vio F, Kain J, Uauy R (2001). Nutrition transition in Latin America: the case of Chile. Nutr Rev.

[35] Instituto Nacional de Estadística y Censos-Costa Rica (INEC) (2002). IX Censo de Población 2000.

[36] Borge C (2007). Consulta en los territorios indígenas del pacífico de Costa Rica.

[37] Kappelle M, Castro M, Acevedo H, González L, Monge H (2002). Ecosistemas del Área de Conservación Osa (ACOSA).

[38] Ministerio de Planificación Nacional y Política Económica (MIDEPLAN). Plan Nacional de desarrollo de los pueblos indígenas de Costa Rica. San José, Costa Rica: MIDEPLAN; 2002.

[39] Sierra C, Vartanián D, Polimeni J (2003). Caracterización social, económica y ambiental del área de conservación Osa.

[40] Horna B (1997). El Impacto de la globalización del Mercado del Banano en la Cultura Indoamericana Guaymí-Ngóbe de la República de Panamá. In: America latina en el umbral del siglo XXI. Encuentro de Latinoamericanistas Españoles, VI.

[41] Royo A. La ocupación del Pacífico Sur Costarricense por parte de la Compañía Bananera (1938–1984). Diálogos Revista Electrónica de Historia (UCR-CR). 2004;4(2):2–30.

[42] Quinlan M (2005). Considerations for Collecting Freelists in the Field: Examples from Ethobotany. Field Methods.

[43] Weller SC, Romney AK (1988). Systematic Data Collection.

[44] Weller SC, Russell Bernard H (1998). Structured Interviewing and Questionnaire Construction. Handbook of Methods in Cultural Anthropology.

[45] Borgatti SP (1996). ANTHROPAC.

[46] Pelto PJ, Pelto GH (1978). Anthropological Research: The Structure of Inquiry.

[47] Bernard R (2005). Research Methods in Anthropology.

[48] Blaikie N (2010). Designing social research: the logic of anticipation.

[49] Martin GJ (2004). Ethnobotany.

[50] Newing H (2011). Conducting Research in Conservation: Social Science Methods and Practice.

[51] Romney AK, Weller SC, Batchelder WH (1986). Culture as consensus: A theory of culture and informant accuracy. Am Anthropol.

[52] Smith J (1993). Using ANTHROPAC 3.5 and a spreadsheet to compute a free-list salience index. Cult. Anthropol. Methods.

[53] van Vliet N, Mertz O, Heinimann A, Langanke T, Pascual U, Schmook B (2012). Trends, drivers and impacts of changes in swidden cultivation in tropical forest-agriculture frontiers: A global assessment. Glob Environ Chang.

[54] Fox J, Yayoi Fujita F, Ngidang D, Peluso P, Potter L, Sakuntaladewi N (2009). Policies, Political-Economy, and Swidden in Southeast Asia. Hum Ecol.

[55] Ross N (2002). Cognitive aspects of intergenerational change: Mental models, cultural change, and environmental behavior among the Lacandón Maya of southern Mexico. Hum Org.

[56] Steward A (2007). Nobody farms here anymore: Livelihood diversification in the Amazonian community of Carvao, a historical perspective. Agric Hum Values.

[57] Virtanen PK (2012). Indigenous Youth in Brazilian Amazonia. Changing Lived Worlds.

[58] Kuhnlein HV, Receveur O (1996). Dietary change and traditional food systems of indigenous peoples. Annu Rev Nutr.

[59] Johns T, Powell B, Maundu P, Eyzaguirre PB (2013). Agricultural biodiversity as a link between traditional food systems and contemporary development, social integrity and ecological health. J Sci Food Agric.

[60] Maffi L, Woodley E (2010). Biocultural Diversity Conservation: A Global Sourcebook.

[61] Nabhan GP, St Antoine S, Kellert SR, Wilson EO (1993). The loss of floral and faunal story: The extinction of experience. The biophilia hypothesis.

[62] Ellen R, Murray T (1999). Forest knowledge, forest transformation: political contingency, historical ecology and the renegotiation of nature in central Seram. Transforming the Indonesian uplands. Marginality, power and production.

[63] Ellen R, Parkes P, Bicker A (eds.). Indigenous Environmental Knowledge and its Transformations. Critical Anthropological Perspectives. Amsterdam: Harwood academic publishers; 2000.

[64] Reyes-García V, Kightley E, Ruiz-Mallén I, Fuentes-Peláez N, Demps K, Huanca T (2010). Schooling and local environmental knowledge: Do they complement or substitute each other?. Int J Educ Dev.

[65] Zent S, Ellen R, Lycett SJ, Johns SE (2013). Processual perspectives on traditional environmental knowledge: continuity, erosion, transformation, innovation. Understanding cultural transmission in anthropology: a critical synthesis.

[66] The Nature Conservancy (TNC) (2001). Planificacion para la conservacion de sitios. Informe para el Area de Conservación Osa (ACOSA).

[67] Baltodano J, Rojas I, Guadrón ME (2009). Los Ngöbes y el bosque.

[68] Hammel BE, Grayum MH, Herrera C, Zamora N (eds.). Manual de Plantas de Costa Rica: Volumen II. Gimnospermas y Monocotiledóneas (Agavaceae - Musaceae). St. Louis: Missouri Botanical Garden Press; 2003.

[69] Hammel BE, Grayum MH, Herrera C, Zamora N (eds.). Manual de Plantas de Costa Rica: Volumen III. Monocotiledóneas (Orchidaceae - Zingiberaceae). St. Louis: Missouri Botanical Garden Press; 2003.

[70] Hammel BE, Grayum MH, Herrera C, Zamora N (eds.). Manual de Plantas de Costa Rica: Volumen VI. Dicotiledóneas (Haloragaceae - Phytolaccaceae). St. Louis: Missouri Botanical Garden Press; 2007.

[71] Hammel BE, Grayum MH, Herrera C, Zamora N (eds.). Manual de Plantas de Costa Rica: Volumen V. Dicotiledóneas (Clusiaceae - Gunneraceae). St. Louis: Missouri Botanical Garden Press; 2010.

[72] Hammel BE, Grayum MH, Herrera C, Zamora N (eds.). Manual de Plantas de Costa Rica: Volumen VII. Dicotiledóneas (Picramniaceae - Rutaceae). St. Louis: Missouri Botanical Garden Press; 2014.

[73] Dahlgren RMT, Clifford HT, Yeo PF (1985). The families of the monocotyledons: structure, evolution and taxonomy.

[74] Cronquist A (1981). An integrated system of classification of flowering plants.

